# Editorial: Biomarkers: precision nutrition in chronic diseases

**DOI:** 10.3389/fnut.2023.1257125

**Published:** 2023-07-24

**Authors:** Zhenjun Zhu, Yu-Long Li, Shuang Song

**Affiliations:** ^1^Department of Food Science and Engineering, College of Science and Engineering, Jinan University, Guangzhou, China; ^2^Department of Emergency Medicine, University of Nebraska Medical Center, Omaha, NE, United States; ^3^School of Food Science and Technology, National Engineering Research Center of Seafood, Dalian Polytechnic University, Dalian, China

**Keywords:** precision nutrition, biomarkers, chronic diseases, dietary intervention, multi-omics, bioinformatics, gut microbiota, metabolite

Chronic diseases, such as cardiovascular diseases, diabetes, cancer, and arthritis, constitute significant causes of morbidity and mortality on a global scale, with their prevalence steadily rising across all age groups, genders, and ethnicities (1). Growing evidence indicates that precision nutrition plays a crucial role in the prevention and management of chronic diseases, garnering recognition as a key area of focus for health research in the next decade (2, 3). Nonetheless, one of the primary challenges in precision nutrition lies in the accurate and reliable assessment of foods and nutrients, particularly with regards to complex foods and macromolecules. Additionally, there is a need to determine how these foods and nutrients impact the health and disease status of individuals.

Promisingly, robust evidence strongly supports the use of biomarkers as an intermediary tool that effectively establishes a connection between precision nutrition and chronic diseases (4). This connection facilitates the objective assessment of food consumption and provides precise determinations of the biological effects of complex foods and ingredients (5, 6). Despite these advancements, our current understanding of how precision nutrition regulates biomarkers to prevent chronic diseases with individual variations is still in its infancy. The molecular mechanisms that underlie the involvement of key biomarkers in chronic diseases remain inadequately elucidated, necessitating comprehensive and extensive research efforts to bridge this knowledge gap. Therefore, the objective of this Research Topic is to gather the latest research that uncovers the role of key biomarkers in chronic diseases and explores how precision nutrition can modulate this process in diverse populations. By investigating the interaction between precision nutrition, biomarker discovery, and chronic diseases, we can gain valuable insights into the implementation of precision nutrition approaches for the effective prevention and management of chronic diseases.

For this Research Topic, a total of 72 manuscripts were received, out of which 25 have been published. Such a high number of submissions indicates that the topic is currently a prominent research focus and a significant area of interest. Presented below is a brief overview of the 25 published articles.

Several novel potential biomarkers for various chronic diseases have been identified in this Research Topic. Among them, a single substance serves as the biomarker, such as 25-hydroxyvitamin D for coronary heart disease (Zhang H. et al.), retinol for non-alcoholic fatty liver (Niu et al.), fluorescent advanced glycation end products for type 2 diabetes (Liu R. et al.), and branched-chain amino acids for moyamoya disease (Zeng et al.). Additionally, the ratio of different substances has been proposed as the biomarker. Hyun et al. and Xia et al. found that the creatine to cystatin C (Cr/CysC) ratio and the albumin to globulin ratio can serve as non-invasive biomarkers for the prognosis of chronic kidney disease and urological cancers, respectively. Notably, Gao et al. have found that the Cr/CysC ratio could also be a potential biomarker for osteoporosis. Furthermore, Cai et al. identified the geriatric nutritional risk index as a potential marker for stroke in elderly hypertensive patients, and Ávila et al. found the level of phosphorus combined with albumin as a potential marker for all-cause mortality and cardiovascular mortality. Intriguingly, Zhang Y. et al. demonstrated that differentially expressed genes related to ferroptosis could be employed as new biomarkers for identifying ischemic stroke and guiding therapeutic interventions. These discovered biomarkers provide an important theoretical foundation for the prevention and management of related chronic diseases, as well as contribute to the understanding of the pathogenesis underlying various chronic diseases.

This Research Topic encompasses some studies focusing on the application of precision nutrition to prevent chronic diseases based on biomarkers, which are typical physiological indicators of such conditions. For instance, studies by Tao et al., Yang et al., Li et al., and Fang et al. demonstrate that dietary supplementation of functional factors, namely anthocyanin, glutamine, Vitamin K, and fatty acids, can respectively alleviate heart failure, high salt-induced hypertension, vascular calcification, and bone mineral loss. However, it is important to exercise caution regarding the dosage of dietary functional factors as excessive fatty acid intake can contribute to metabolic diseases. Other articles highlight the positive effects of complex foods on various chronic diseases. For example, Huo et al. demonstrate that dietary fruit consumption improves functional constipation, Shahdadian et al. endorse a plant-based diet for managing metabolic syndrome, Hevilla et al. propose specific oral nutritional supplements (ONS) to address inflammation/oxidation, and Zamani et al. suggest saffron for mitigating cardiovascular diseases. Notably, Hevilla et al. also observe a synergistic effect of probiotic supplementation in conjunction with specific ONS on inflammation/oxidation. Additionally, Huang et al. demonstrate that combined training, involving resistance training along with high-intensity interval training or moderate-intensity continuous training, is beneficial for non-alcoholic fatty liver disease treatment. In addition to these dietary strategies for preventing or treating chronic diseases, Liu T.-h. et al. mention that dietary patterns also contribute to the onset of such conditions, such as a high-salt diet exacerbating the intestinal aging process. These dietary functional factors and complex foods present effective preventive measures for managing chronic diseases. However, the specific mechanisms responsible for the regulation of these biomarkers have not been thoroughly explored, highlighting the need for further research in this area.

During the process of mining biomarkers, it is crucial to consider the individual differences among research subjects. Jáni et al. demonstrate that some factors such as birth length, puberty onset, and visceral fat levels can collectively influence the biological aging process, accounting for 21% of the observed variation. Furthermore, the findings reviewed by Wuni et al. suggest that additional factors, such as individual socio-economic status and architectural environment, also influence the same process. Consequently, it becomes imperative to enhance the reliability of biomarker mining using cutting-edge techniques such as bioinformatics technology. Cole et al. employed genetic heritability as a tool to evaluate the accuracy of dietary questionnaire variables in their research, thereby bolstering the credibility of biomarker mining in dietary studies. Additionally, Barbe et al. effectively circumvented the impact of individual variability on nutritional intervention in immune and metabolic health by utilizing metabolomics-based clustering methods. Similarly, Xu et al. employed a bidirectional Mendelian randomization study to establish hypothetical causal relationships between circulating vitamin D and estimated bone mass, plasma triglycerides, and total cholesterol. This approach helped avoid inconsistencies in results caused by the presence of confounding factors. Despite the advances made by these approaches in bolstering the credibility of biomarker mining, the process remains susceptible to numerous unpredictable confounders. Therefore, further advancements are required to enhance the causal verification of biomarkers through animal experiments and human clinical studies.

In conclusion, this Research Topic has contributed to a better understanding of additional biomarkers associated with chronic diseases and the measures of precision nutrition interventions. Furthermore, the topic addresses factors that may interfere with biomarker identification and presents effective techniques for circumventing these challenges. However, the causal verification of biomarkers and the molecular mechanisms underlying their precise nutritional regulation still require further clarification, which will be the central focus of the articles included in Volume II of this Research Topic.

## Author contributions

ZZ prepared the first draft. Y-LL and SS critically reviewed and edited it. All authors approved the submitted version.
